# Supplementary material for: Multiplexed analysis of the secretin-like GPCR-RAMP interactome
Source: Sci Adv. 2019 Sep 18;5(9):eaaw2778. doi: 10.1126/sciadv.aaw2778 (PMC6750928; doi:10.1126/sciadv.aaw2778)
Supplement: http://advances.sciencemag.org/cgi/content/full/5/9/eaaw2778/DC1 [file supp_5_9_eaaw2778__index.html]

Science Advances | Science AdvancesAAASSearchScience AdvancesMenu

## Supplementary Materials

**This PDF file includes:**

- Supplementary Methods
- Fig. S1. Coexpression of GPCR clusters with RAMPs and the position of selected GPCRs on the phylogenetic tree.
- Fig. S2. Validation of anti-epitope tag mAbs to capture and detect engineered RAMPs and GPCRs.
- Fig. S3. Validation of Abs used to capture RAMPs.
- Fig. S4. Analysis of anti-GPCR Ab cross-reactivity.
- Fig. S5. Detection of GPCR-RAMP complexes following capture by all anti-GPCR Abs.
- Fig. S6. Statistical validation of GPCR-RAMP SBA datasets.
- Fig. S7. Detection of CALCRL-RAMP2 interactions in cell membranes using PLA.
- Table S1. The ID of the bead coupled to each specific Ab, the source of the Ab, and the product code.
- Table S2. Statistical significances of complex formation between each GPCR and RAMP complex pair reported as *P* values.
- Table S3. Overall statistic for GPCR-RAMP complex formation.
- Table S4. Statistical metrics used to compare GPCR-RAMP complex formation datasets.
- References (*41*–*43*)

Download PDF

**Files in this Data Supplement:**

- Adobe PDF - aaw2778\_SM.pdf
