# Supplementary material for: Multiplexed analysis of the secretin-like GPCR-RAMP interactome
Source: Sci Adv. 2019 Sep 18;5(9):eaaw2778. doi: 10.1126/sciadv.aaw2778 (PMC6750928; doi:10.1126/sciadv.aaw2778)
Supplement: Download PDF [file aaw2778_SM.pdf]

Science Advances
AAAS

advances.sciencemag.org/cgi/content/full/5/9/eaaw2778/DC1

Supplementary Materials for

Multiplexed analysis of the secretin-like GPCR-RAMP interactome

Emily Lorenzen, Tea Dodig-Crnković, Ilana B. Kotliar, Elisa Pin, Emilie Ceraudo, Roger D. Vaughan, Mathias Uhlèn, Thomas Huber, Jochen M. Schwenk*, Thomas P. Sakmar*

*Corresponding author. Email: jochen.schwenk@scilifelab.se (J.M.S.); sakmar@rockefeller.edu (T.P.S.)

Published 18 September 2019, *Sci. Adv.* **5**, eaaw2778 (2019)
DOI: 10.1126/sciadv.aaw2778

This PDF file includes:

Supplementary Methods
Fig. S1. Coexpression of GPCR clusters with RAMPs and the position of selected GPCRs on the phylogenetic tree.
Fig. S2. Validation of anti-epitope tag mAbs to capture and detect engineered RAMPs and GPCRs.
Fig. S3. Validation of Abs used to capture RAMPs.
Fig. S4. Analysis of anti-GPCR Ab cross-reactivity.
Fig. S5. Detection of GPCR-RAMP complexes following capture by all anti-GPCR Abs.
Fig. S6. Statistical validation of GPCR-RAMP SBA datasets.
Fig. S7. Detection of CALCRL-RAMP2 interactions in cell membranes using PLA.
Table S1. The ID of the bead coupled to each specific Ab, the source of the Ab, and the product code.
Table S2. Statistical significances of complex formation between each GPCR and RAMP complex pair reported as *P* values.
Table S3. Overall statistic for GPCR-RAMP complex formation.
Table S4. Statistical metrics used to compare GPCR-RAMP complex formation datasets.
References (*41–43*)

Supplementary Materials

Supplementary Methods

Co-expression analysis: RNA-seq data from 53 tissues provided by 544 donors, with a total of 8555 samples, were downloaded from gtexportal.org (GTEx_Analysis_v6p_RNA-seq_RNA-SeQCv1.1.8_gene_rpkm.gct.gz) *(41)*. Downloaded data was provided as reads per kilobase per million mapped reads (RPKM). Only samples passing quality control were included in the dataset. Read counts and RPKM values were produced with RNA-SeqC; importantly, reads were mapped to a single gene (see gtexportal.org documentation for more information). For quantile normalization, gene expression across an individual sample was fit to the averaged distribution observed across samples. Prior to implementing spearman correlation analysis, the median normalized expression per tissue was calculated to account for differences in the number of samples per tissue type. Spearman correlation coefficient was calculated for each RAMP/non-olfactory GPCR pair. GPCR clusters were assigned as per Fredriksson et al *(42)*.

Statistical comparison of data sets: The data obtained using anti-GPCR capture Abs was compared to the data obtained using the epitope-tag capture methods. Based upon the data set for GPCR-RAMP complexes derived from the epitope-tag capture Abs, a matrix of hypothetical outcomes for the data set derived from the anti-GPCR Ab capture strategy was constructed. To compare the two matrices, the results were converted to binary score matrices (0,1) and the two matrices (epitope-tag Ab capture *versus* direct Ab capture). A Z-score threshold of 1.645 was applied for the anti-GPCR Ab data set of 1.645, which corresponds to a confidence interval of 95% for a single-tailed test (fig. S5). The threshold used to convert the summarized and normalized epitope tag data to binary form was increased by an interval of 0.125 (table S2). The

following metrics were plotted as a function of the threshold used to convert the epitope tag data to a binary matrix: (1) overall percent agreement (P_0), (2) the percent of hits from the epitope tag data that are also found in the anti-GPCR Ab data (sensitivity), (3) the percent of non-hits from the epitope tag data that are also non-hits in the anti-GPCR Ab data (specificity), (4) the probability of a positive result in the anti-GPCR Ab data also being a positive in the epitope tag data (positive predictive value), (5) the probability of a negative result in the anti-GPCR Ab data also being a negative in the epitope tag data (negative predictive value) and (6) the similarity of the two data sets (Jaccard Index). The formulas used are listed in table S4.

Supplementary Figures

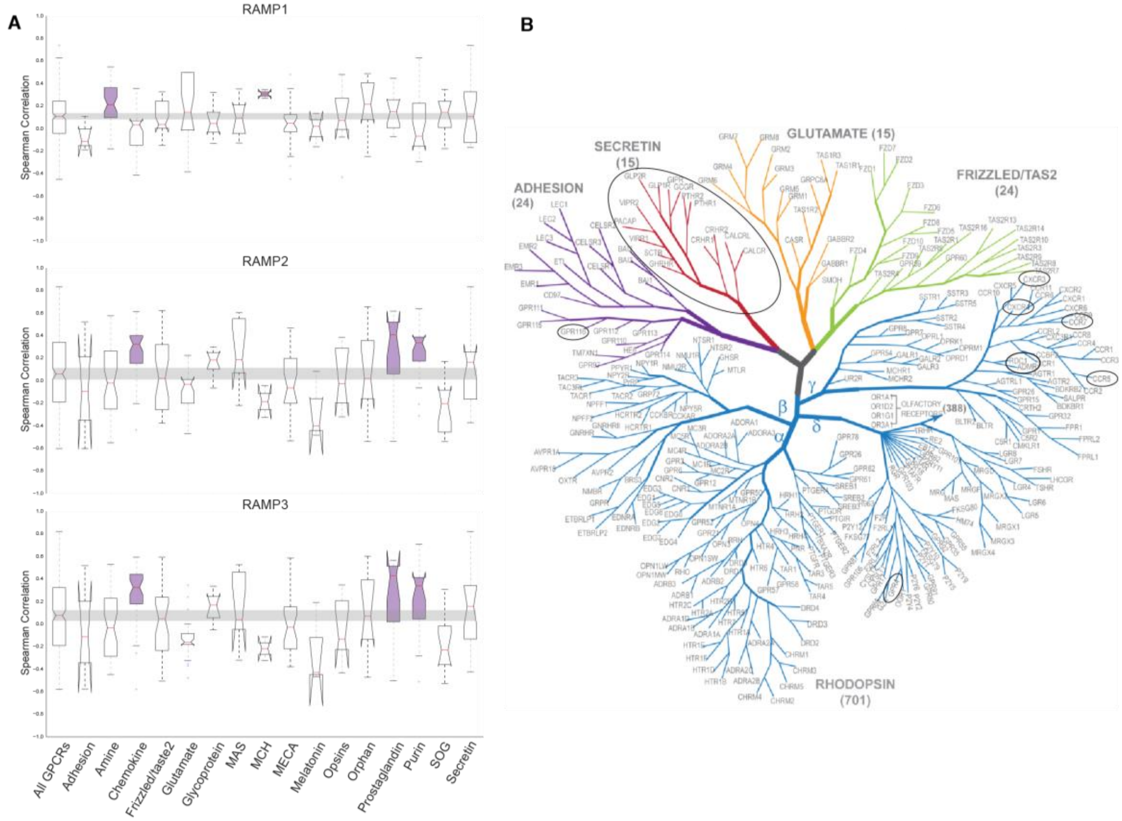

Fig. S1. Coexpression of GPCR clusters with RAMPs and the position of selected GPCRs on the phylogenetic tree. (**A**) Co-expression of GPCRs clusters with each RAMP in comparison to all GPCRs. Boxplot of spearman correlation coefficients across 53 human tissues between RAMP-GPCR pairs. Notches indicate 95% confidence intervals of the median and the grey bars indicate the 95% confidence interval of the median for all GPCRs. Receptor clusters with significantly higher co-expression than all GPCRs are highlighted with purple. Abbreviations: MCH, melanin-concentrating hormone; MECA, melanocortin, endothelial differentiation, cannabinoid, and adenosine; SOG, somatostatin, opioid, and galanin. (**B**) GPCR phylogenetic tree highlighting the receptors tested for complex formation with RAMPs *(42, 43)*. Circled GPCR names indicate that the receptor was included in this study. In the phylogenetic tree,

ACKR3 is referred to as RDC1, GPR182 is ADMR, ADGRF5 is GPR116, and ADCYAP1R1 is PACAP.

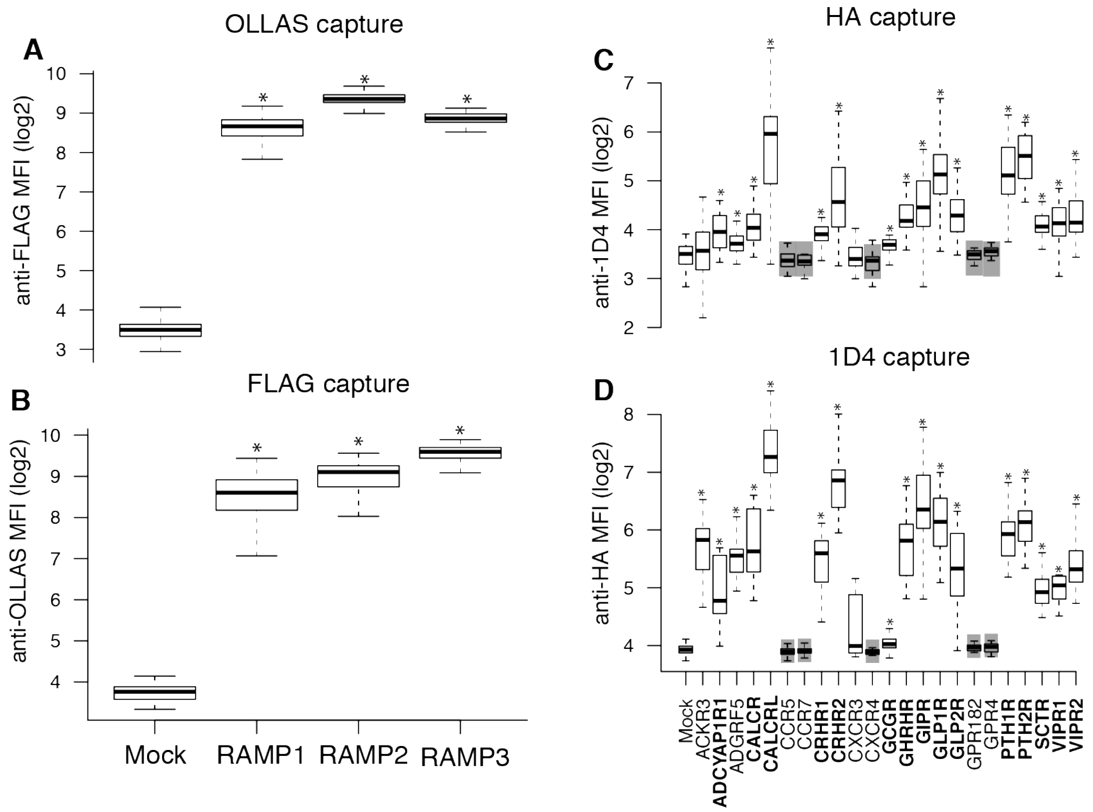

Fig. S2. Validation of anti-epitope tag mAbs to capture and detect engineered RAMPs and GPCRs. Lysates from cells transfected with either epitope-tagged RAMP only (FLAG and OLLAS) or co-expressed with a GPCR were incubated with the SBA, which included beads harboring anti-FLAG and anti-OLLAS mAbs. Each RAMP was (**A**) captured with anti-OLLAS mAb bead and detected with PE-conjugated anti-FLAG mAb, or (**B**) captured with anti-FLAG mAb bead and detected with a PE-conjugated anti-OLLAS mAb. Lysates from cells transfected with either epitope-tagged GPCR only (HA and/or 1D4) or co-expressed with a RAMP were incubated with the SBA, which included beads harboring anti-HA or anti-1D4 mAbs. Each GPCR was (**C**) captured with anti-HA mAb bead and detected with a PE-conjugated anti-1D4 mAb, or (**D**) captured with anti-1D4 mAb bead and detected with a PE-conjugated anti-HA mAb. Grey boxes around the occasional data set indicate that the GPCR does not have both engineered epitope tags, and thus would not be expected to show signal in this experiment. The

labels in bold correspond to secretin-like GPCRs. Data are median fluorescence intensity (MFI) and representative of at least nine experiments performed in duplicate. * indicates that signal was significantly higher ($p \leq 0.5$) than mock (Ordinary one-way ANOVA, Dunnett's multiple comparisons test).

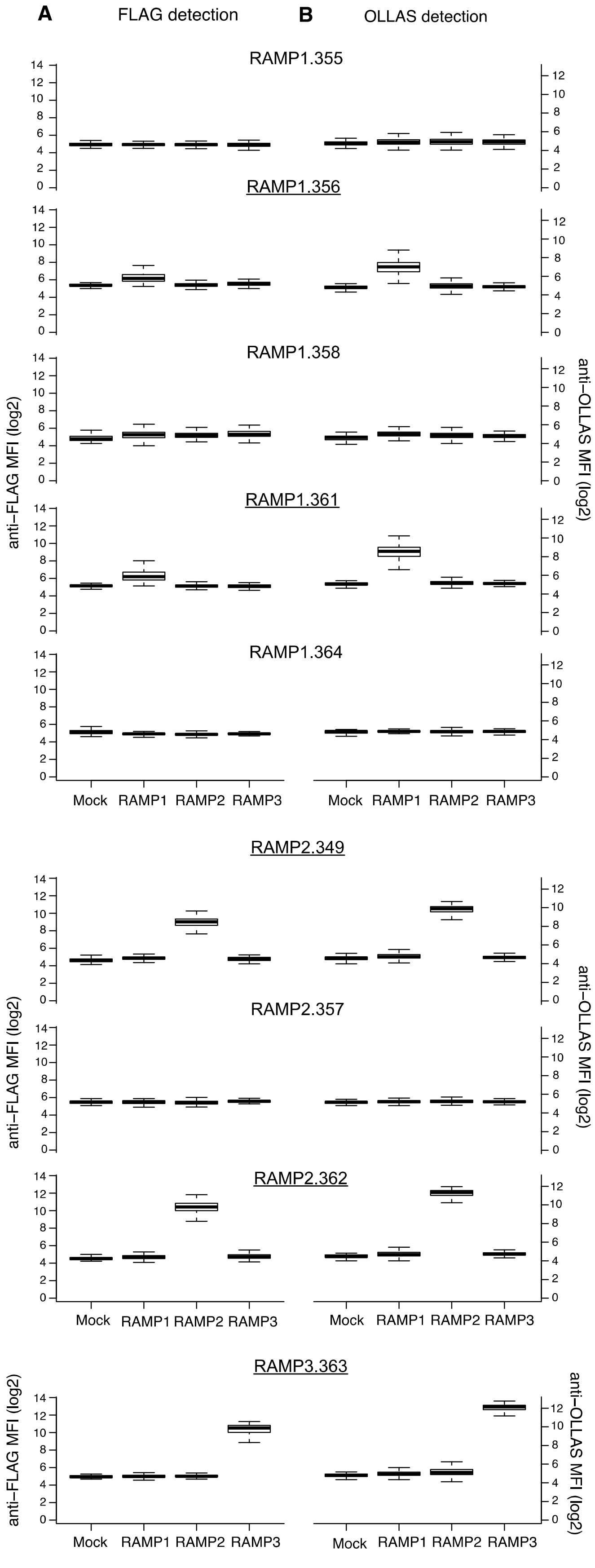
A
FLAG detection
B
OLLAS detection
RAMP1.355
RAMP1.356
RAMP1.358
RAMP1.361
RAMP1.364
anti−FLAG MFI (log2)
anti−OLLAS MFI (log2)
Mock
RAMP1
RAMP2
RAMP3
RAMP2.349
RAMP2.357
RAMP2.362
RAMP3.363

Fig. S3. Validation of Abs used to capture RAMPs. In order to validate anti-RAMP Abs, lysates from cells transfected with each epitope-tagged RAMP construct (FLAG and OLLAS) were incubated with the SBA, which included beads conjugated with nine capture Abs targeting the three RAMPs. (**A**) PE-conjugated anti-FLAG and (**B**) PE-conjugated anti-OLLAS mAbs were used to detect any RAMPs captured by the beads. Data are median fluorescence intensity (MFI) and represent at least 200 experiments, each performed in duplicate. At a statistical significance of $p \leq 0.05$ (Kruskal-Wallis ANOVA), we validated at least one capture Ab for each of the three RAMPs (a total of 5 RAMP capture Abs). Bead ID numbers are listed after each RAMP name and the corresponding Ab name is provided in table S1.

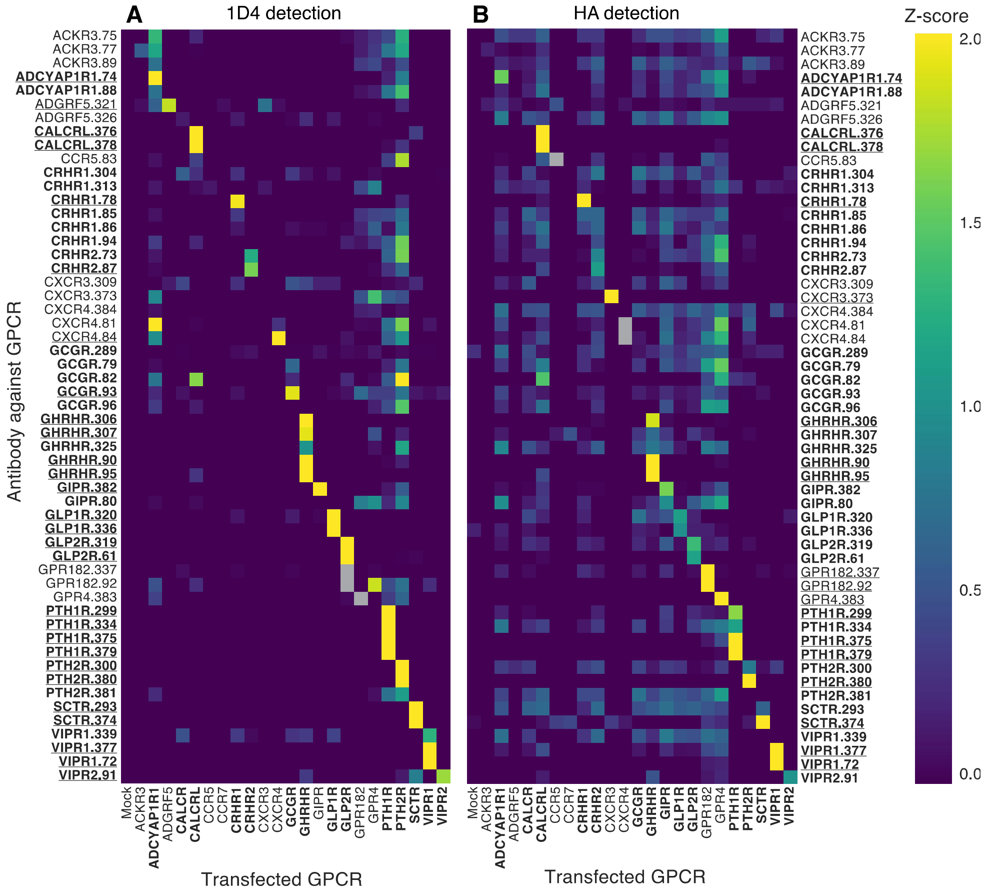

Fig. S4. Analysis of anti-GPCR Ab cross-reactivity. Lysates from cells transfected with each epitope-tagged GPCR construct (HA and 1D4) were incubated with the SBA, which included 55 Abs to 21 GPCRs. (**A**) PE-conjugated anti-1D4 and (**B**) PE-conjugated anti-HA were used to detect any GPCRs captured by the beads. The occasional grey boxes indicate that the GPCR did not have the appropriate epitope tag to be detected. The labels in bold correspond to secretin-like GPCRs and validated Abs are underlined Abs. Heatmaps represent the z-scores of median fluorescence intensity (MFI) and indicates the ability of the GPCR Abs to capture each of the 23 GPCRs used in the study. Data represents the median z-score of at least three experiments performed in duplicate. Bead ID numbers are listed after each GPCR name and the corresponding Ab name is provided in table S1.

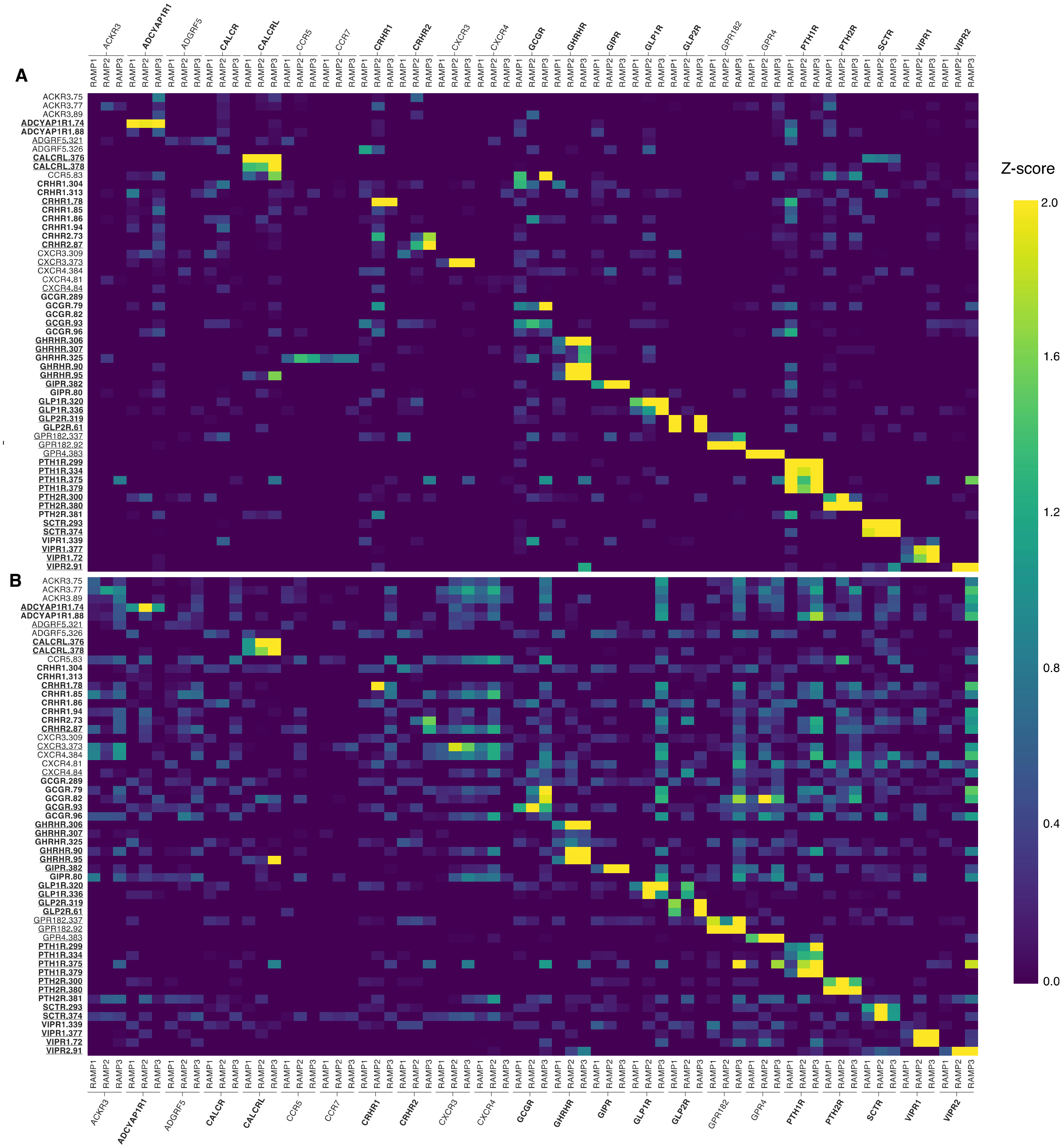

Fig. S5. Detection of GPCR-RAMP complexes following capture by all anti-GPCR Abs. Lysates from cells transfected with each epitope-tagged RAMP construct (FLAG and OLLAS) and co-transfected with each epitope-tagged GPCR construct (HA and 1D4) were incubated with the SBA, which included 55 Abs to the 21 GPCRs included in this study. Complexes were captured in multiplex fashion using the anti-GPCR Ab beads. The presence of a GPCR-RAMP

complex was detected using (**A**) PE-conjugated OLLAS mAb, or (**B**) PE-conjugated anti-FLAG mAb. The labels in bold correspond to secretin-like GPCRs and validated Abs are underlined. The heatmap displays the Z-score of median fluorescence intensity (MFI) and represents at least three experiments performed in duplicate. Bead ID numbers are listed after each GPCR name and the corresponding Ab name is provided in table S1.

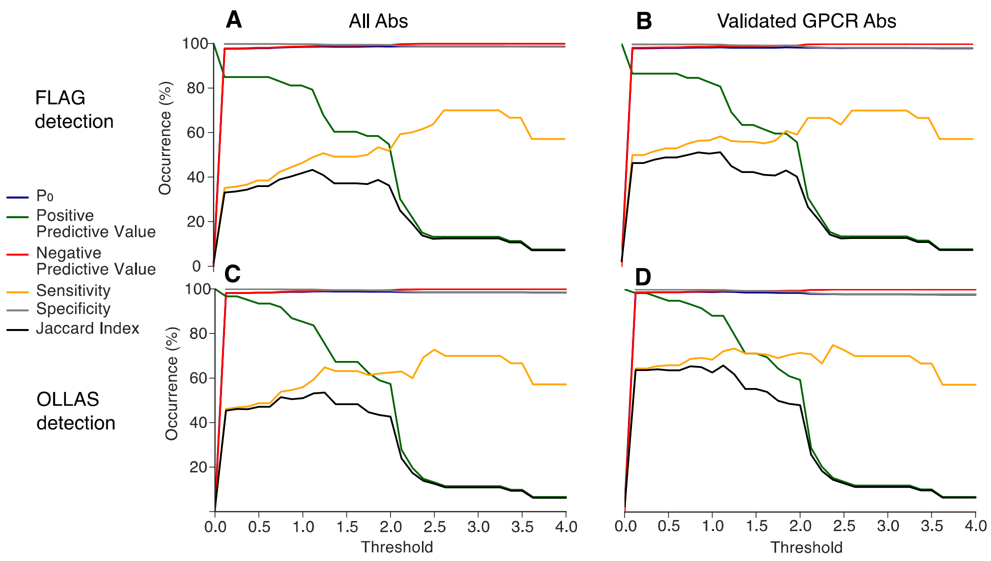

Fig. S6. Statistical validation of GPCR-RAMP SBA datasets. Data from the capture of the GPCR-RAMP complexes with anti-epitope mAbs were compared with data obtained from the GPCR-RAMP complexes captured using anti-GPCR Abs. PE-conjugated anti-FLAG was used to detect GPCR-RAMP complexes captured using (**A**) all anti-GPCR Abs or (**B**) validated anti-GPCR Abs. Alternatively, PE-conjugated anti-OLLAS mAb was used to detect GPCR-RAMP complexes captured using (**C**) all anti-GPCR Abs or (**D**) validated anti-GPCR Abs. The Z-score threshold for the anti-GPCR Ab data was set at 1.645. P_0 (blue), positive predictive value (green), negative predictive value (red), sensitivity (yellow), specificity (grey), and Jaccard Index (black) are plotted as a function of increasing threshold for the interaction results using epitope tags for capture and detection (table S2). Supplementary Materials and Methods shows the formulas and metrics used and provides a narrative description of each of the statistical terms. For example, the Jaccard Index represents the overall agreement of the positive results in both data sets and indicates at which thresholds the agreement is maximized.

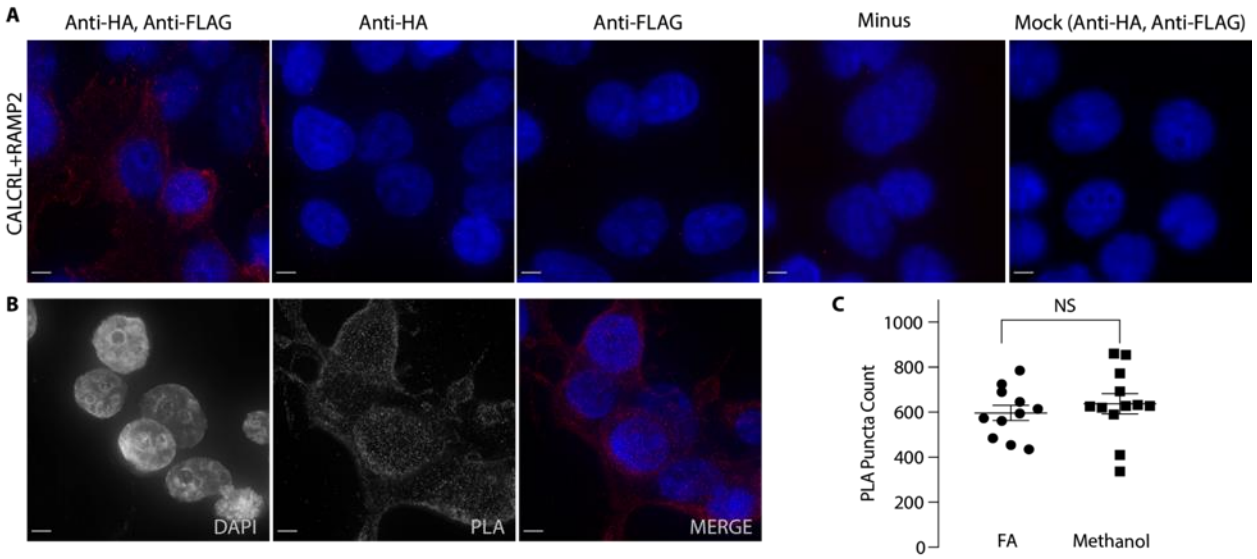

Fig. S7. Detection of CALCRL-RAMP2 interactions in cell membranes using PLA. Cells co-transfected with epitope-tagged GPCR and RAMP2 then incubated with anti-HA and anti-FLAG Abs. PLA was then carried out to detect GPCR-RAMP2 interactions. (**A**) Representative images of PLA performed on CALCRL+RAMP2 co-transfected cells using Ab detection as noted. Images show maximum projection of Z-stack, which is the maximum signal intensity for each channel at each point across all slices in the Z-stack. (**B**) Representative PLA images showing greyscale split-channel view of a Z-stack maximum projection for cells co-transfected with CALCRL+RAMP2 and treated with both primary Abs. The merge is presented in color. Scale bars, 5μm for both (A) and (**C**). Blue = DAPI, red = PLA puncta. (C) PLA puncta counts per cell for cells co-transfected with CALCRL+RAMP2, fixed with either FA or methanol and subjected to PLA. Data are from at least two experiments performed with at least five replicates. Significance determined by two-tailed P-test (P=0.4763, NS = not significant).

Table S1. The ID of the bead coupled to each specific Ab, the source of the Ab, and the product code. The labels in bold correspond to secretin-like GPCRs. Underlined product codes indicate validated Abs.

Bead ID	Protein Name	Antibody Source	Product Code
61	**GLP2R**	HPA	HPA027929
72	**VIPR1**	HPA	HPA046516
73	**CRHR2**	HPA	HPA046683
74	**ADCYAP1R1**	HPA	HPA030739
75	ACKR3	HPA	HPA049718
77	ACKR3	HPA	HPA032003
78	**CRHR1**	HPA	HPA063352
79	**GCGR**	HPA	HPA066333
80	**GIPR**	HPA	HPA068054
81	CXCR4	HPA	HPA068321
82	**GCGR**	HPA	HPA071228
83	CCR5	HPA	HPA070587
84	CXCR4	HPA	HPA051623
85	**CRHR1**	HPA	HPA055287
86	**CRHR1**	HPA	HPA071484
87	**CRHR2**	HPA	HPA073345
88	**ADCYAP1R1**	HPA	HPA073908
89	ACKR3	HPA	HPA057492
90	**GHRHR**	**HPA**	HPA077545
91	**VIPR2**	**HPA**	HPA062707
92	**GPR182**	**HPA**	HPA027037
93	**GCGR**	**HPA**	HPA057075
94	**CRHR1**	**HPA**	HPA046066
95	**GHRHR**	**HPA**	HPA070884
96	**GCGR**	**HPA**	HPA074345
293	**SCTR**	**HPA**	HPA007312
299	**PTH1R**	**HPA**	HPA007978
300	**PTH2R**	**HPA**	HPA010534
304	**CRHR1**	**HPA**	HPA032018
306	**GHRHR**	**HPA**	HPA034645
307	**GHRHR**	**HPA**	HPA034644
309	CXCR3	HPA	HPA045942
313	**CRHR1**	**HPA**	HPA052441
319	**GLP2R**	**HPA**	HPA064671
320	**GLP1R**	**HPA**	HPA065175

Bead ID	Protein Name	Antibody Source	Product Code
321	ADGRF5	HPA	HPA065251
325	**GHRHR**	HPA	HPA068576
326	ADGRF5	HPA	HPA068796
334	**PTH1R**	HPA	HPA075879
336	**GLP1R**	HPA	HPA077988
337	GPR182	HPA	HPA027037
343	Empty	N/A	N/A
339	**VIPR1**	HPA	HPA026777
349	RAMP2	HPA	HPA064452
350	rabbit IgG	Bethyl	P120
355	RAMP1	Abcam	ab156575
356	RAMP1	HPA	HPA010654
357	RAMP2	HPA	HPA052020
358	RAMP1	HPA	HPA057814
359	OLLAS	In house	N/A
360	FLAG	Sigma	F3165
361	RAMP1	RnD	AF6428
362	RAMP2	RnD	AF6427
363	RAMP3	RnD	AF4875
364	RAMP1	Santa	sc-11379
365	mouse	Bio Rad	PMP01X
366	1D4	In house	N/A
367	HA	Biolegend	16B12
373	CXCR3	HPA	HPA003189
374	**SCTR**	HPA	HPA007269
375	**PTH1R**	HPA	HPA007491
376	**CALCRL**	HPA	HPA007586
377	**VIPR1**	HPA	HPA007588
378	**CALCRL**	HPA	HPA008070
379	**PTH1R**	HPA	HPA007978
380	**PTH2R**	HPA	HPA010534
381	**PTH2R**	HPA	HPA010655
382	**GIPR**	HPA	HPA017428
383	GPR4	HPA	HPA019207
384	CXCR4	HPA	HPA027832

Table S2. Statistical significances of complex formation between each GPCR and RAMP complex pair reported as *P* values. The data sets are from GPCR-RAMP complexes capture and detected by mAbs targeting epitope tags on the GPCR and RAMP constructs. Statistical significance of signal between mock transfected cell lysates and lysates from cells co-transfected with each dual-tagged RAMP construct plus each dual or single-tagged GPCR construct. (Ordinary one-way ANOVA, Dunnett's multiple comparisons test). N/A indicates that the GPCR does not have the epitope tag to be captured or detected with the corresponding Ab. Secretin-like receptors are shown in bold.

	Interaction with RAMP1							
Capture Ab	1D4	1D4	HA	HA	OLLAS	OLLAS	FLAG	FLAG
Detection Ab	FLAG	OLLAS	FLAG	OLLAS	1D4	HA	1D4	HA
ACKR3	0.857	0.804	1.000	1.000	0.999	1.000	1.000	1.000
**ADCYAP1R1**	0.204	0.025	1.000	1.000	0.001	1.000	0.000	1.000
ADGRF5	0.392	0.017	1.000	1.000	0.706	1.000	0.907	1.000
**CALCR**	0.079	0.002	1.000	1.000	0.986	1.000	0.994	1.000
**CALCRL**	0.000	0.000	0.000	0.000	0.000	0.000	0.000	0.000
CCR5	1.000	1.000	N/A	N/A	1.000	N/A	1.000	N/A
CCR7	1.000	1.000	N/A	N/A	1.000	N/A	1.000	N/A
**CRHR1**	0.999	0.999	1.000	1.000	1.000	1.000	1.000	1.000
**CRHR2**	0.927	0.808	0.999	1.000	0.994	1.000	0.999	0.999
CXCR3	0.999	0.969	1.000	1.000	0.999	1.000	1.000	1.000
CXCR4	0.999	0.999	1.000	1.000	1.000	1.000	1.000	1.000
**GCGR**	0.016	0.000	1.000	0.999	0.904	1.000	0.855	1.000
**GHRHR**	0.280	0.069	0.999	1.000	0.158	1.000	0.491	0.999
**GIPR**	0.000	0.000	0.447	0.999	0.053	0.994	0.028	0.603
**GLP1R**	0.000	0.000	0.170	0.821	0.000	0.999	0.000	0.884
**GLP2R**	0.000	0.000	0.944	1.000	0.000	0.995	0.000	0.494
GPR182	N/A	N/A	0.000	0.275	N/A	0.023	N/A	0.000
GPR4	N/A	N/A	0.000	0.440	N/A	0.003	N/A	0.000
**PTH1R**	0.000	0.000	0.690	0.721	0.000	0.995	0.000	0.864
**PTH2R**	0.000	0.000	0.005	0.302	0.000	0.879	0.000	0.244
**SCTR**	0.032	0.000	1.000	0.999	0.000	0.975	0.000	1.000
**VIPR1**	0.630	0.181	1.000	0.999	0.073	1.000	0.106	1.000
**VIPR2**	0.003	0.087	0.999	1.000	0.632	1.000	0.611	1.000

	Interaction with RAMP2							
Capture Ab	1D4	1D4	HA	HA	OLLAS	OLLAS	FLAG	FLAG

Detection Ab	FLAG	OLLAS	FLAG	OLLAS	1D4	HA	1D4	HA
ACKR3	0.000	0.000	0.988	1.000	0.907	1.000	0.070	0.999
**ADCYAP1R1**	0.060	0.021	1.000	1.000	0.062	1.000	0.000	1.000
ADGRF5	0.000	0.000	1.000	0.999	0.987	1.000	0.977	1.000
**CALCR**	0.000	0.000	0.958	0.985	0.994	1.000	0.456	0.999
**CALCRL**	0.000	0.000	0.000	0.000	0.000	0.000	0.000	0.000
CCR5	0.408	0.153	N/A	N/A	0.999	N/A	0.999	N/A
CCR7	0.995	0.418	N/A	N/A	0.975	N/A	0.999	N/A
**CRHR1**	0.001	0.000	0.994	0.999	0.999	1.000	0.988	1.000
**CRHR2**	0.471	0.115	0.041	1.000	0.986	1.000	0.883	0.869
CXCR3	0.988	0.782	0.995	0.995	1.000	1.000	1.000	0.995
CXCR4	0.999	0.999	1.000	1.000	1.000	1.000	1.000	1.000
**GCGR**	0.000	0.000	1.000	1.000	0.999	1.000	0.501	1.000
**GHRHR**	0.000	0.000	0.709	1.000	0.956	1.000	0.210	0.999
**GIPR**	0.000	0.000	0.000	0.021	0.000	0.941	0.000	0.816
**GLP1R**	0.000	0.000	0.009	0.556	0.003	0.996	0.000	0.827
**GLP2R**	0.000	0.000	0.021	0.982	0.000	0.994	0.000	0.984
GPR182	N/A	N/A	0.000	0.000	N/A	0.000	N/A	0.000
GPR4	N/A	N/A	0.000	0.000	N/A	0.000	N/A	0.000
**PTH1R**	0.000	0.000	0.095	0.966	0.000	0.989	0.000	0.712
**PTH2R**	0.000	0.000	0.065	0.090	0.000	0.254	0.000	0.648
**SCTR**	0.001	0.000	0.999	0.925	0.000	0.944	0.000	0.999
**VIPR1**	0.030	0.002	0.995	0.995	0.016	1.000	0.000	0.999
**VIPR2**	0.000	0.000	0.995	0.984	0.167	1.000	0.002	0.999

	Interaction with RAMP3							
Capture Ab	1D4	1D4	HA	HA	OLLAS	OLLAS	FLAG	FLAG
Detection Ab	FLAG	OLLAS	FLAG	OLLAS	1D4	HA	1D4	HA
ACKR3	0.001	0.000	1.000	1.000	0.999	0.999	0.999	0.999
**ADCYAP1R1**	0.024	0.000	1.000	1.000	0.005	0.981	0.190	0.999
ADGRF5	0.016	0.000	1.000	1.000	0.820	0.999	0.999	1.000
**CALCR**	0.000	0.000	1.000	0.983	0.281	0.996	0.923	0.999
**CALCRL**	0.000	0.000	0.000	0.063	0.000	0.000	0.000	0.000
CCR5	0.999	0.995	N/A	N/A	1.000	N/A	1.000	N/A
CCR7	0.999	0.551	N/A	N/A	0.994	N/A	0.999	N/A
**CRHR1**	0.123	0.000	1.000	1.000	0.999	1.000	0.999	1.000
**CRHR2**	0.008	0.000	0.999	0.977	0.157	0.995	0.817	0.586
CXCR3	0.885	0.039	0.999	0.999	1.000	1.000	1.000	1.000
CXCR4	1.000	0.999	1.000	1.000	1.000	1.000	1.000	1.000
**GCGR**	0.000	0.000	1.000	1.000	0.297	0.999	0.776	1.000
**GHRHR**	0.000	0.000	0.999	0.999	0.017	0.925	0.147	0.740
**GIPR**	0.000	0.000	0.961	0.999	0.016	0.063	0.193	0.736
**GLP1R**	0.000	0.000	0.964	0.372	0.040	0.306	0.017	0.522
**GLP2R**	0.000	0.000	1.000	0.999	0.000	0.050	0.000	0.569

GPR182	N/A	N/A	0.000	0.000	N/A	0.000	N/A	0.000
GPR4	N/A	N/A	0.000	0.000	N/A	0.000	N/A	0.000
**PTH1R**	0.000	0.000	0.809	0.633	0.000	0.451	0.000	0.034
**PTH2R**	0.000	0.000	0.350	0.141	0.000	0.078	0.000	0.145
**SCTR**	0.003	0.000	1.000	0.995	0.000	0.955	0.000	0.984
**VIPR1**	0.000	0.000	0.999	0.860	0.001	0.965	0.043	0.999
**VIPR2**	0.000	0.000	0.933	0.926	0.000	0.862	0.001	0.911

Table S3. Overall statistic for GPCR-RAMP complex formation. P-values from table S2 of <0.0001 were assigned 4, <0.001 a 3, <0.01 a 2, and <0.05 a 1. The values were summed and divided by the number of capture and detection pairs that we expected to be capable of measuring the relevant complex. For dual-tagged GPCRs, we divided by eight and for single-tagged GPCRs we divided by four to obtain a normalized value. Secretin-like receptors are shown in bold. * indicates that the complex formation was confirmed by PLA and † that complex formation was not confirmed by PLA.

	RAMP1	RAMP2	RAMP3	**Previously Published RAMP interaction**
ACKR3	0	1.125	0.75	Unknown
**ADCYAP1R1**	1	0.75	0.75	Unknown
ADGRF5	0.125	0.875	0.625	Unknown
**CALCR**	0.25	1	1	RAMP1, 2 and 3 (*3*)
**CALCRL**	4	4*	3.5	RAMP1, 2 and 3 (*1*, *2*)
CCR5	0	0	0	Unknown
CCR7	0	0	0	Unknown
**CRHR1**	0	0.625	0.375	RAMP2 (*10*, *12*)
**CRHR2**	0	0	0.75	None (*12*)
CXCR3	0	0†	0.25	Unknown
CXCR4	0	0	0	Unknown
**GCGR**	0.625	1	2	Unknown
**GHRHR**	0	2	1.125	RAMP2 (*8*, *9*, *11*)
**GIPR**	1.125	2.375	1.125	None (*9*)
**GLP1R**	1.75	1.875	1.25	None (*8–10*)
**GLP2R**	2	2	2	None (*9*)
GPR182	2.25	2.25*	4	Unknown
GPR4	2.5	3.25*	4	Unknown

**PTH1R**	2	2*	2.125	RAMP2 (*9*)
**PTH2R**	2.25	2	2.125	RAMP3 (*9*)
**SCTR**	1.625	2	1.75	RAMP3 (*7*)
**VIPR1**	0	1.125	1.25	RAMP1, 2 and 3 (*9*)
**VIPR2**	0.25	1.25	1.625	RAMP1, 2, and 3 (*10*)

Table S4. Statistical metrics used to compare GPCR-RAMP complex formation datasets. Formulas for the metrics and information derived from each formula are also listed. TP, true positive; TN, true negative; FP, false positive; TP, true positive.

Metric	Formula	Information
P_0	(TP+TN)/(TP+TN+FP+FN)	Overall percent agreement
Sensitivity	TP/(TP+FN)	Percent of all positives from epitope beads that the anti-GPCR beads detect
Specificity	TN/(TN+FP)	Percent of all negatives from epitope beads that the anti-GPCR beads detect
Positive Predictive Value	TP/(TP+FP)	Probability of being a positive from epitope beads if anti-GPCR beads says positive
Negative Predictive Value	TN/(TN+FN)	Probability of being a negative from epitope beads if anti-GPCR beads says negative
Jaccard Index	TP/(TP+FP+FN)	Similarity of positives between the epitope beads and the anti-GPCR beads
